# Supplementary material for: Artesunate regulates malignant progression of breast cancer cells via lncRNA TUG1/miR-145-5p/HOXA5 axis
Source: PLoS One. 2025 Aug 4;20(8):e0329490. doi: 10.1371/journal.pone.0329490 (PMC12321065; doi:10.1371/journal.pone.0329490)
Supplement: Attachment — Submitted filename: Response to Reviewers 6.2.pdf [file pone.0329490.s002.pdf]

PONE-D-24-47234R1
Artesunate Regulates the Malignant Progression of Breast Cancer Cells via the lncRNA TUG1/miR-145-5p/HOXA5 Axis
PLOS ONE

Dear Dr. Yang,

Thank you for submitting your manuscript to PLOS ONE. After careful consideration, we feel that it has merit but does not fully meet PLOS ONE’s publication criteria as it currently stands. Therefore, we invite you to submit a revised version of the manuscript that addresses the points raised during the review process.

Please submit your revised manuscript by Jun 13 2025 11:59PM. If you will need more time than this to complete your revisions, please reply to this message or contact the journal office at plosone@plos.org. When you're ready to submit your revision, log on to https://www.editorialmanager.com/pone/ and select the 'Submissions Needing Revision' folder to locate your manuscript file.

Please include the following items when submitting your revised manuscript:
A rebuttal letter that responds to each point raised by the academic editor and reviewer(s). You should upload this letter as a separate file labeled 'Response to Reviewers'.
A marked-up copy of your manuscript that highlights changes made to the original version. You should upload this as a separate file labeled 'Revised Manuscript with Track Changes'.
An unmarked version of your revised paper without tracked changes. You should upload this as a separate file labeled 'Manuscript'.
If you would like to make changes to your financial disclosure, please include your updated statement in your cover letter. Guidelines for resubmitting your figure files are available below the reviewer comments at the end of this letter.

If applicable, we recommend that you deposit your laboratory protocols in protocols.io to enhance the reproducibility of your results. Protocols.io assigns your protocol its own identifier (DOI) so that it can be cited independently in the future. For instructions see: https://journals.plos.org/plosone/s/submission-guidelines#loc-laboratory-protocols. Additionally, PLOS ONE offers an option for publishing peer-reviewed Lab Protocol articles, which describe protocols hosted on protocols.io. Read more information on sharing protocols at https://plos.org/protocols?utm_medium=editorial-email&utm_source=authorletters&utm_campaign=protocols.

We look forward to receiving your revised manuscript.
Kind regards,

Manikkam Rajalakshmi
Academic Editor
PLOS ONE

Journal Requirements:

Please review your reference list to ensure that it is complete and correct. If you have cited papers that have been retracted, please include the rationale for doing so in the manuscript text, or remove these references and replace them with relevant current references. Any changes to the reference list should be mentioned in the rebuttal letter that accompanies your revised manuscript. If you need to cite a retracted article, indicate the article's retracted status in the References list and also include a citation and full reference for the retraction notice.

Reviewers' comments:

Reviewer's Responses to Questions
Comments to the Author

Reviewer #1: All comments have been addressed

Reviewer #2: All comments have been addressed

2. Is the manuscript technically sound, and do the data support the conclusions?

The manuscript must describe a technically sound piece of scientific research with data that supports the conclusions. Experiments must have been conducted rigorously, with appropriate controls, replication, and sample sizes. The conclusions must be drawn appropriately based on the data presented.

Reviewer #1: Partly

Reviewer #2: Yes

3. Has the statistical analysis been performed appropriately and rigorously?

Reviewer #1: N/A

Reviewer #2: Yes

4. Have the authors made all data underlying the findings in their manuscript fully available?

The PLOS Data policy requires authors to make all data underlying the findings described in their manuscript fully available without restriction, with rare exception (please refer to the Data Availability Statement in the manuscript PDF file). The data should be provided as part of the manuscript or its supporting information, or deposited to a public repository. For example, in addition to summary statistics, the data points behind means, medians and variance measures should be available. If there are restrictions on publicly sharing data—e.g. participant privacy or use of data from a third party—those must be specified.

Reviewer #1: Yes

Reviewer #2: Yes

5. Is the manuscript presented in an intelligible fashion and written in standard English?

PLOS ONE does not copyedit accepted manuscripts, so the language in submitted articles must be clear, correct, and unambiguous. Any typographical or grammatical errors should be corrected at revision, so please note any specific errors here.

Reviewer #1: Yes

Reviewer #2: Yes

6. Review Comments to the Author

Please use the space provided to explain your answers to the questions above. You may also include additional comments for the author, including concerns about dual publication, research ethics, or publication ethics. (Please upload your review as an attachment if it exceeds 20,000 characters)

Dear Reviewer,

We sincerely appreciate the time and effort you have dedicated to reviewing our manuscript. Your thorough analysis and constructive feedback have provided invaluable guidance in refining our research, ensuring it is scientifically sound and impactful. We are grateful for your professionalism and your insightful comments, which have significantly contributed to improving our study on target identification and drug discovery for breast cancer.

We fully acknowledge the gaps and concerns you have highlighted. Specifically, we recognize that relying exclusively on in vitro models

(MCF7, HCC1395) limits the translational relevance of our findings. The absence of in vivo validation, such as xenograft models or patient-derived xenografts (PDX), is a critical limitation, and we concur that demonstrating ART's efficacy in suppressing tumor growth, metastasis, and toxicity in living systems is essential. Additionally, we understand the importance of pharmacokinetic/pharmacodynamic (PK/PD) data to confirm whether the proposed TUG1/miR-145-5p/HOXA5 axis functions in vivo.

At the same time, we acknowledge that no single research article can comprehensively answer all scientific questions regarding such a complex issue. Science is a continuous journey of discovery, requiring ongoing experiments and validations to strengthen conclusions. Your comments have provided excellent direction for designing and executing future experiments, which we wholeheartedly intend to pursue.

However, the time and funding required for the additional experiments you suggested—particularly in vivo studies and expanded mechanistic investigations—are substantial. As a relatively small research team, we rely on continuous applications for funding projects, and securing additional support hinges upon achieving incremental milestones, including the publication of our current findings. Without a tangible demonstration of progress through publication, obtaining further resources to conduct additional experiments would be challenging.

Therefore, we kindly request that our revision focus on refining and optimizing our current experimental approach. Your insights on improving our methodology and ensuring the robustness of our present results would be instrumental in helping us secure the necessary funding to further explore and validate our findings. We deeply appreciate your support and understanding in this regard.

Once again, thank you for your invaluable feedback and for helping us enhance the quality and impact of our research. We look forward to incorporating your recommendations and advancing this important work.

Please see below the response to each comment.

Reviewer #1: 1. Lack of In Vivo Validation
- Critical Gap: The study relies entirely on in vitro models (MCF7, HCC1395). While these are standard, the absence of in vivo data (e.g., xenograft models, PDX) severely limits translational relevance.

- Specific Concerns:
- ART's efficacy in suppressing tumor growth, metastasis, and toxicity in living systems remains unverified.
- No pharmacokinetic/pharmacodynamic (PK/PD) data to confirm whether the proposed TUG1/miR-145-5p/HOXA5 axis operates in vivo .
- Recommendations:
- Include xenograft experiments using ART-treated mice to validate tumor suppression and pathway modulation.
- Measure ART and metabolite levels (e.g., DHA) in serum/tissue to correlate dosing with effects.

Response: Thank you very much for the suggestion. I agree with you that in vivo experiment would be the next steps to further validate the therapeutic effect of the molecule. At this point, we are a very small research group, and the funding is extremely limited. Also, the waiting list for such animal experiment is very long in our institutional laboratory, and it could take up to 6 months before all the ethical approval and animal orders to be completed. Thanks again for your understanding that we are facing challenges in many ways for such tasks. However, there's no doubt that you provided detailed and scientifically sound suggestions for the experiments needed to further verify current findings. Thank you so much!

2. Clinically Irrelevant Dosing
- Critical Issue: ART concentrations (30 – 480 μM) far exceed pharmacologically achievable levels in humans. Typical plasma concentrations after standard malaria dosing (2 – 4 mg/kg) are ~1 – 10 μM.
- Specific Concerns:
- High doses (e.g., 480 μM) risk off-target toxicity and may not reflect therapeutic windows.
- No justification for dose selection or comparison to prior preclinical/clinical studies.
- Recommendations:
- Justify dose ranges using PK data (e.g., cite studies where ART inhibited cancer cells at ≤10 μM).
- Test lower, clinically relevant doses (1 – 20 μM) to confirm pathway-specific effects.
- Discuss potential toxicity (e.g., hepatotoxicity, bone marrow suppression) at high doses.

Response: We appreciate your concern regarding the appropriate concentrations of artesunate used in our experiment. The concentration range of 30–480 μM employed in our study is well-supported by existing literature and aligns with previous research investigating artesunate's anti-cancer effects.

Several studies have utilized artesunate concentrations up to 200 µg/mL (Wen, Lijuan et al. “Artesunate promotes G2/M cell cycle arrest in MCF7 breast cancer cells through ATM activation.” Breast cancer (Tokyo, Japan) vol. 25,6 (2018): 681-686. doi:10.1007/s12282-018-0873-5), which corresponds to 520.83 µM, based on its molecular weight. Additionally, prior cancer research has explored artesunate concentrations as high as 500 µM (Wang, Ning et al. “Artesunate inhibits proliferation and invasion of mouse hemangioendothelioma cells in vitro and of tumor growth in vivo.” Oncology letters vol. 14,5 (2017): 6170-6176. doi:10.3892/ol.2017.6986), demonstrating its potential efficacy in cancer treatment.

Crystal violet staining assay

Cells were seeded onto the 6-well plates at 5×10^5 cells/well. After cell attachment, cells were incubated at different concentrations of ART (0, 12.5, 25, 50, 100, and 200 µg/ml) for 48 h. The cells were then rinsed twice with pre-cooled PBS and fixed with 4% paraformaldehyde for 30 min. After removal of the fixation solution, fixed cells were stained with crystal violet for 10 min. Images were obtained using an inverted microscope.

Growth inhibition assay. An MTT assay was used to assess cell proliferation. The EOMA cells ($1x10^5$/ml) were seeded onto 96-well plates. Artesunate was diluted in 1 ml 5% sodium bicarbonate solution, shaken for 2-3 min producing 0, 100, 200, 300, 400 and 500 µg/ml concentrations and then 100 µl was added to the EOMA cells in each well. At 0, 24, 48 and 72 h, an equal volume of MTT solution was added to each well and cultured for another 4 h. The incubation conditions in this assay were all 37°C in an atmosphere containing 5% CO_2. MTT-treated cells were fixed with 150 µl dimethyl sulfoxide for 30 min at room temperature and then assayed with an Evolution™ 201/220 UV-Vis spectrophotometer at 490 nm.

It is important to note that the appropriate concentration of artesunate in anti-cancer research remains a subject of ongoing debate. However, it is widely acknowledged that the concentration required for cancer treatment is significantly higher than that used for malaria therapy. Clinical studies have investigated artesunate at doses of 45 mg/kg (Artemisinin and Its Derivatives in Cancer Care. Canadian College of Naturopathic Medicine, Jan. 2024, ccnm.edu.), far exceeding the standard malaria dosing of 2–4 mg/kg.

A phase I dose escalation trial evaluated safety, pharmacokinetics, and response rates of IV artesunate in patients with advanced refractory solid tumors.[24] Nineteen patients were administered artesunate on days 1 and 8 on a 21-day cycle with dose escalation levels of 8, 12, 18, 25, 34, and 45 mg/kg. 18/19 participants were analyzed for toxicity. The maximum tolerated dose (MTD) was determined to be 18 mg/kg, after 1/6 patients experienced dose limiting toxicities (DLT) at both the 12 mg/kg and 18 mg/kg doses, whereas 2/2 patients had a DLT at 25 mg/kg. DLTs included neutropenic fever, hypersensitivity reaction, liver function abnormality, and nausea/vomiting. Many non DLTs were recorded including anemia, neutropenia, gastrointestinal side effects (e.g. diarrhea, constipation, nausea/vomiting), elevations in LFTs, fatigue, loss of appetite, electrolyte imbalances, hypoalbuminemia, arthralgias, dizziness, headache, and cough. Pharmacokinetic analysis demonstrated a short half-life of ART (Tmax=5 min; T1/2=7–8 min) and rapid metabolism by esterase-catalyzed hydrolysis to its active metabolite, DHA. DHA had a Tmax of 20min, and median half-life of 30 minutes to 3.5 hrs which increased with dose. 15/19 patients were evaluated for response; 4/15 had stable disease, there were no partial or complete responses.

Furthermore, one of the key advantages of Chinese herbal medicine, including artesunate, is its relatively low toxicity as a natural product, which supports its potential for therapeutic applications in oncology.

In summary, our study represents an initial step in the drug discovery process, and there is substantial scientific evidence supporting the concentrations used in our experiment. We sincerely appreciate the reviewer's careful and professional evaluation of our work.

3. Narrow Mechanistic Focus

- Critical Oversight: The study focuses solely on the TUG1/miR-145-5p/HOXA5 axis, ignoring ART' s well-documented roles in ferroptosis, autophagy, and ROS generation.
- Specific Concerns:
- No experiments to rule out contributions from other pathways (e.g., GPX4 for ferroptosis, LC3B for autophagy).
- Overlooked crosstalk between WNT/β-catenin and other oncogenic pathways (e.g., STAT3, AKT).
- Recommendations:
- Perform RNA-seq/proteomics to identify additional ART-regulated pathways.
- Inhibit ferroptosis (e.g., ferrostatin-1) or autophagy (e.g., chloroquine) to test if ART' s effects persist.

Response: We sincerely appreciate your thoughtful insights and recommendations regarding the mechanistic scope of our study. Your observations are highly valuable, and we fully acknowledge the necessity of broadening our experimental approach to explore additional ART-regulated pathways beyond the TUG1/miR-145-5p/HOXA5 axis. We agree that evaluating ART's roles in ferroptosis, autophagy, and ROS generation is crucial for developing a more comprehensive understanding of its therapeutic effects. Specifically, investigating GPX4's involvement in ferroptosis and LC3B's role in autophagy could provide deeper mechanistic clarity. Additionally, we recognize the importance of assessing the potential crosstalk between WNT/β-catenin and other oncogenic pathways such as STAT3 and AKT, as suggested. While we acknowledge the necessity of conducting RNA-seq and proteomics to identify broader ART-regulated pathways and validating these findings through inhibition studies (e.g., ferrostatin-1 for ferroptosis, chloroquine for autophagy), we are currently facing financial constraints that limit our ability to execute these additional experiments at this stage. The cost associated with high-throughput analyses and targeted inhibition assays presents a challenge within our current funding framework. Nonetheless, we are committed to incorporating these experiments in our future research. We are actively seeking additional financial support and grant opportunities that will enable us to expand the scope of our study as recommended. Once sufficient funding is secured, we will proceed with the suggested experimental approaches to ensure a more comprehensive investigation of ART's mechanistic actions. Thank you for your constructive feedback. We genuinely appreciate your insightful suggestions and look forward to strengthening our research accordingly.

4. Limited Model Diversity
- Issue: Only two breast cancer cell lines (MCF7, HCC1395) were tested, both luminal/subtypes. Triple-negative (TNBC) or HER2+ models are absent.
- Recommendation: Include TNBC lines (e.g., MDA-MB-231) to assess broader applicability.

Response: Thank you for your valuable feedback regarding model diversity in our study. We fully agree that incorporating Triple-Negative Breast Cancer (TNBC) and HER2+ models, such as MDA-MB-231, would enhance the applicability of our findings. Expanding our study to include these subtypes will provide deeper insights into the broader therapeutic potential of our approach. However, due to financial constraints, acquiring and maintaining additional cell lines and resources is currently challenging. Despite this, we are actively seeking further funding opportunities to support the expansion of our research. Once additional funding is secured, we will prioritize the suggested experiments to strengthen the robustness of our conclusions. We appreciate your constructive recommendations and remain committed to implementing them in our future work.

5. Data Transparency
- Issue: Uncropped Western blot images and raw qPCR data are not clearly accessible via the Dryad link.
- Recommendation: Provide annotated, full-length blot images and raw Ct values in SI.

Response: Please see supplementary information for complete set of uncropped western blot images and qPCR data. We will later update it on Dryad.

Critical Revisions Required
1. Prioritize In Vivo Experiments: At minimum, include pilot xenograft data to support translational claims.
2. Re-evaluate Dosing Strategy: Align ART concentrations with clinically achievable levels and provide pharmacokinetic rationale.
3. Broaden Mechanistic Scope: Address alternative pathways (e.g., ferroptosis) to confirm the TUG1 axis is the primary driver.

Reviewer #2: (No Response)

7. PLOS authors have the option to publish the peer review history of their article (what does this mean?). If published, this will include your full peer review and any attached files.

If you choose “no”, your identity will remain anonymous but your review may still be made public.

Do you want your identity to be public for this peer review? For information about this choice, including consent withdrawal, please see our Privacy Policy.

Reviewer #1: Yes: Ahmed I. Abd El Maksoud

Reviewer #2: No

[NOTE: If reviewer comments were submitted as an attachment file, they will be attached to this email and accessible via the submission site. Please log into your account, locate the manuscript record, and check for the action link "View Attachments". If this link does not appear, there are no attachment files.]

While revising your submission, please upload your figure files to the Preflight Analysis and Conversion Engine (PACE) digital diagnostic tool, https://pacev2.apexcovantage.com/. PACE helps ensure that figures meet PLOS requirements. To use PACE, you must first register as a user. Registration is free. Then, login and navigate to the UPLOAD tab, where you will find detailed instructions on how to use the tool. If you encounter any issues or have any questions when using PACE, please email PLOS at figures@plos.org. Please note that Supporting Information files do not need this step.

In compliance with data protection regulations, you may request that we remove your personal registration details at any time. (Remove my information/details). Please contact the publication office if you have any questions.
