# Supplementary material for: Artesunate regulates malignant progression of breast cancer cells via lncRNA TUG1/miR-145-5p/HOXA5 axis
Source: PLoS One. 2025 Aug 4;20(8):e0329490. doi: 10.1371/journal.pone.0329490 (PMC12321065; doi:10.1371/journal.pone.0329490)
Supplement: Attachment — Submitted filename: Response to reviewers 4.4.docx [file pone.0329490.s001.docx]

Journal requirements:

When submitting your revision, we need you to address these additional requirements.

1. Please ensure that your manuscript meets PLOS ONE's style requirements, including those for file naming. The PLOS ONE style templates can be found at

<https://journals.plos.org/plosone/s/file?id=wjVg/PLOSOne_formatting_sample_main_body.pdf> and <https://journals.plos.org/plosone/s/file?id=ba62/PLOSOne_formatting_sample_title_authors_affiliations.pdf>
RE: Thank you for your professional advice. We have made corresponding changes as required, and the content now complies with the specific formatting requirements.

2.  PLOS ONE now requires that authors provide the original uncropped and unadjusted images underlying all blot or gel results reported in a submission’s figures or Supporting Information files. This policy and the journal’s other requirements for blot/gel reporting and figure preparation are described in detail at https://journals.plos.org/plosone/s/figures#loc-blot-and-gel-reporting-requirements and https://journals.plos.org/plosone/s/figures#loc-preparing-figures-from-image-files. When you submit your revised manuscript, please ensure that your figures adhere fully to these guidelines and provide the original underlying images for all blot or gel data reported in your submission. See the following link for instructions on providing the original image data: https://journals.plos.org/plosone/s/figures#loc-original-images-for-blots-and-gels.  
 In your cover letter, please note whether your blot/gel image data are in Supporting Information or posted at a public data repository, provide the repository URL if relevant, and provide specific details as to which raw blot/gel images, if any, are not available. Email us at [plosone@plos.org](mailto:plosone@plos.org) if you have any questions.
RE: Thank you for your professional advice. All the original data have been provided along with detailed explanations. Please refer to the “Raw_images” file.

3. Please amend either the title on the online submission form (via Edit Submission) or the title in the manuscript so that they are identical.
RE: Thank you for your professional advice. Thank you for your professional advice. We have modified the title and made it consistent across the text.

4.  Please include a caption for Figure 1A, Figure 2A & B, Figure 3A, Figure 4A & B, Figure 5A & B.

RE: Thank you for your professional advice. We have added the results of another cell line to the results figures and have included corresponding legend explanations below the results, updating the previous legend descriptions. Please refer to the results section in the Manuscript.

5. Thank you for stating the following financial disclosure:

 [This work was supported by the Training Project of Clinical Medical Professionals (2023), ZF2023065, provided by the government of Hebei Province.]. Please state what role the funders took in the study.  If the funders had no role, please state: "The funders had no role in study design, data collection and analysis, decision to publish, or preparation of the manuscript." If this statement is not correct you must amend it as needed. Please include this amended Role of Funder statement in your cover letter; we will change the online submission form on your behalf.

RE: Thank you for your professional advice. This statement is correct and valid, and the appropriate declaration has been provided.

Reviewers' comments:

Reviewer's Responses to Questions

**Comments to the Author**

1. Is the manuscript technically sound, and do the data support the conclusions?

The manuscript must describe a technically sound piece of scientific research with data that supports the conclusions. Experiments must have been conducted rigorously, with appropriate controls, replication, and sample sizes. The conclusions must be drawn appropriately based on the data presented.

Reviewer #1: Yes

Reviewer #2: Yes

2. Has the statistical analysis been performed appropriately and rigorously?

Reviewer #1: Yes

Reviewer #2: Yes

3. Have the authors made all data underlying the findings in their manuscript fully available?

The [PLOS Data policy](http://track.editorialmanager.com/CL0/http://www.plosone.org/static/policies.action%23sharing/1/010f019536613acf-1663e915-de07-4821-a194-1d2656418e62-000000/7ee7WzEba2ITL929N4Ejzri0sR1j_nAbpsxA5A1BNS8=199) requires authors to make all data underlying the findings described in their manuscript fully available without restriction, with rare exception (please refer to the Data Availability Statement in the manuscript PDF file). The data should be provided as part of the manuscript or its supporting information, or deposited to a public repository. For example, in addition to summary statistics, the data points behind means, medians and variance measures should be available. If there are restrictions on publicly sharing data—e.g. participant privacy or use of data from a third party—those must be specified.

Reviewer #1: Yes

Reviewer #2: No

4. Is the manuscript presented in an intelligible fashion and written in standard English?

PLOS ONE does not copyedit accepted manuscripts, so the language in submitted articles must be clear, correct, and unambiguous. Any typographical or grammatical errors should be corrected at revision, so please note any specific errors here.

Reviewer #1: Yes

Reviewer #2: Yes

5. Review Comments to the Author

 Please use the space provided to explain your answers to the questions above. You may also include additional comments for the author, including concerns about dual publication, research ethics, or publication ethics. (Please upload your review as an attachment if it exceeds 20,000 characters)

Reviewer #1:

Review of Manuscript PONE-D-24-47234: Artesunate Regulates the Malignant Progression of Breast Cancer Cells via the lncRNA TUG1/miR-145-5p/HOXA5 Axis
Overall Assessment:
This manuscript presents a comprehensive study on the potential anti-cancer effects of artesunate on breast cancer cells, focusing on the lncRNA TUG1/miR-145-5p/HOXA5 axis. The study employs a variety of in vitro assays to explore the proposed pathway, providing valuable insights into the molecular mechanisms underlying artesunate's action. However, there are several areas that require improvement and clarification to enhance the manuscript's quality and impact.
Major Comments:
1. Background and Context:
- The introduction effectively sets the stage by highlighting the challenges in breast cancer treatment and the potential of traditional Chinese medicine, specifically artesunate. However, it would be beneficial to include more recent references to underscore the current state of research in this area.
RE: Thank you for your professional input, updated references on breast cancer and artesunate have been added as per specific request, which can be seen in the preface content under the revised version.
2. Methodology:
- The methods section is detailed and provides a clear understanding of the experimental procedures. However, it would be helpful to include a flowchart or diagram to illustrate the experimental design and timeline, especially for the drug stimulation and plasmid transfection experiments.

RE: Thank you for your professional advice, we have mapped out the flowchart according to the specific groupings and the experiments carried out have been experimented in order and placed in the Experimental Methods section.

- The rationale for choosing specific concentrations of artesunate (30μmol/L, 120μmol/L, and 480μmol/L) should be explained. Are these concentrations based on previous studies or preliminary experiments?

RE: Thank you for your professional advice. Combining with previous research, we conducted a comprehensive literature review before our study. The literature “Artesunate promotes G2/M cell cycle arrest in MCF7 breast cancer cells through ATM activation” shows that the concentrations of artesunate at which the inhibition rate of MCF7 cells reaches 90% are 139.60, 70.00, and 16.45 µg/ml at 24 hours, 48 hours, and 72 hours respectively. Through calculation, the corresponding concentrations in terms of mass-volume fraction are 360 μmol/L, 182 μmol/L, and 41 μmol/L. In the literature “Artesunate-induced Cellular Effects Are Mediated by Specific EPH Receptors and Ephrin Ligands in Breast Carcinoma Cells.”, the concentration of artesunate used to treat MCF7 cells is 100 μmol/L. These experimental evidences suggest that the concentration of artesunate in the range of 40 μmol/L to 380 μmol/L may be effective and has no obvious effect of promoting cell apoptosis. Meanwhile, we also carried out preliminary experiments. First, we detected the IC50 of artesunate on MCF10A and MCF7 cells. The detection was carried out within a relatively broad concentration range (from 0 μmol to 2000 μmol), which helped us determine that the concentration of artesunate used in the experiment was feasible and had no killing effect. Within the range of IC50 and combined with previous experimental data, we selected 30 μmol/L as the lowest effective concentration and 480 μmol/L as the higher effective concentration. With the support of previous data and the reference of preliminary experiments, we can conduct subsequent experiments.

3. Results:
- The results section is well-organized and presents a clear narrative of the findings. However, some figures and tables are not provided in the manuscript, making it difficult to fully assess the data. Ensure that all relevant figures and tables are included.

RE: Thank you for your professional advice. In response, we carefully examined all the result figures, specifically evaluated the data in the result figures, and swapped Figures 4A&B with Figures 4C&D. We then supplemented the remedial data, provided information on the PCR primers, and placed the figure legends below the cited sections as required by the format. Additionally, we provided pictures of the original data. In summary, these are the charts and figures we thought might need to be provided for the review.
- The discussion of the results could be strengthened by comparing the findings with existing literature on artesunate and its effects on cancer cells.
RE: Thank you for your professional advice. We have added the content about artemisinin acting on different types of cancer cells through various mechanisms by citing relevant literature to the discussion section. This provides new ideas for future research and a basis for the expansion of the content of our experiment.

Artesunate induces caspase-dependent apoptosis in breast cancer cells by inhibiting HSP70 protein. In this study, the anti-apoptotic effect of Artesunate is regulated through the LncRNA/miRNA/mRNA cascade reaction, which modulates HOXA5 and subsequently affects the Wnt/β-catenin signaling pathway. Further research is needed to determine which specific caspase protein is involved [25]. Artesunate can promote the degradation of hexokinase 1 (HK1) by targeting it, while simultaneously downregulating the levels of HIF1α and PKM2, thereby inhibiting aerobic glycolysis in esophageal cancer progression. The exacerbation of aerobic glycolysis is a novel metabolic indicator of cancer progression. Whether HOXA5 promotes breast cancer progression by enhancing glycolysis suggests that, based on molecular docking experiments, Artesunate could inhibit the aggravation of malignant phenotypes through LncRNA TUG1-mediated glycolysis suppression [26]. Artesunate inhibits colorectal cancer progression by suppressing reactive oxygen species-induced cellular senescence and autophagy. Whether its effect on breast cancer is mediated through the LncRNA/miRNA/mRNA signaling pathway, influencing the expression of senescence-related proteins and exacerbating endoplasmic reticulum stress, remains unclear [27]. Artesunate can reverse paclitaxel resistance in cancer cells by enhancing lysosomal function. It can also modulate the neurite outgrowth inhibitor protein B receptor to regulate sorafenib resistance in hepatocellular carcinoma. Research on breast cancer drug resistance and the targets mentioned in this study provides new insights [28, 29]. Artesunate induces a novel form of cell death—ferroptosis—in ovarian serous carcinoma, rather than solely relying on apoptosis [30]. Artesunate inhibits melanoma progression by suppressing the STAT3 signaling pathway. In this study, it targets the long non-coding RNA TUG1 to inhibit ovarian cancer growth. The development of comprehensive targets for Artesunate can serve as a new summary [8, 31]. New derivatives of Artesunate can exert cytotoxic effects on various cancer cell lines by inducing GPX4-mediated ferroptosis. Whether the Artesunate used in this study also has multiple derivatives that inhibit breast cancer progression remains unknown [11].
4. Discussion and Conclusions:
- The discussion effectively summarizes the key findings and their implications. However, it would be beneficial to explore the potential clinical applications of artesunate in breast cancer treatment more thoroughly.

RE: Thank you for your professional advice. The potential clinical application of artesunate is highly worthy of discussion. We have conducted an in-depth analysis of the research progress, possibilities, and limitations of its clinical application.
There is still a long way to go for artesunate to be used as a clinical treatment drug for breast cancer. Firstly, there are still relatively few basic studies on artesunate in breast cancer. Artesunate can activate DNA damage and then induce cell-cycle arrest in breast cancer cells [36]. The killing effect of artesunate on breast cancer cell lines is mediated by specific EPH receptors and ephrin ligands [37]. Interestingly, the iron homeostasis maintained by transferrin and its receptor has been thoroughly studied in breast cancer treated with artesunate [38]. Whether the data from the above basic studies are sufficient to support further clinical research remains debatable. Secondly, the development of new formulations is still hindered. The toxicity identification and targeting ability of artesunate nanocapsules still need to be improved [39]. The biological properties of the new camptothecin-artesunate conjugate still need to be identified in terms of pharmacokinetics, pharmacology, and toxicology [40].
- The conclusions are clear and concise, but they could be further supported by highlighting the study's limitations and suggesting avenues for future research.
RE: Thank you for your professional advice. Regarding this point, we have conducted a more extensive discussion on the limitations of the experimental content. Apart from the already established experimental results, it is necessary to explore and study other possibilities.

Although this study has revealed the critical role of lncRNA TUG1 in the anti-tumor effects of Artesunate, the specific molecular mechanisms remain incompletely elucidated. For instance, whether lncRNA TUG1 exerts its functions through interactions with other miRNAs or proteins still requires further investigation. This study focused on the WNT/β-catenin signaling pathway, but whether it intersects with other signaling pathways, and whether it functions through positive or negative feedback regulation, remains unclear. Additionally, whether this study comprehensively represents the signaling pathway network is still a question. The study employed Artesunate at concentrations of 30μM, 120μM, and 480μM for experiments, but whether these doses are clinically feasible in vivo remains uncertain. High doses of Artesunate may induce toxicity in normal cells, potentially limiting its clinical application. Therefore, future research should further optimize the dosage of Artesunate and evaluate its safety and efficacy in vivo. Drug resistance is a common issue in cancer treatment, and future studies should assess whether Artesunate induces resistance and explore strategies to overcome it. The clinical application of Artesunate still faces numerous challenges, such as drug bioavailability, metabolic stability, and administration methods. Future research should explore the clinical translation potential of Artesunate and optimize its drug delivery systems to enhance its anti-tumor efficacy in vivo. Moreover, future studies should consider personalized treatment strategies, such as using genetic testing to screen patient populations sensitive to Artesunate, to improve treatment outcomes.
5. Ethics Statement:
- The ethics statement is brief and does not provide sufficient detail. Given that the study involves cell lines, it would be appropriate to include a statement confirming that the cell lines were obtained from a reputable source and that appropriate ethical guidelines were followed.

RE: Thank you for your professional advice. We purchased commercially available cell lines. This article does not involve animal experiments or human experiments, so the explanation is relatively brief. All the cell lines were used for experiments after being identified by STR and being free of mycoplasma. Below is the STR profiling report for MCF7 and MCF10A.All the cells were purchased from Procell Life Science & Technology Co., Ltd.

6. Data Availability:
- The data availability statement is clear and indicates that all data are fully available without restriction. However, it would be helpful to specify where the data can be accessed, such as a public repository or as supplementary materials.

RE: Thank you for your expert advice. Most of the content in this paper consists of basic experiments, and we have submitted the original images as supplementary materials.

Minor Comments:

1. Formatting and Style:
- There are inconsistencies in formatting and style throughout the manuscript. Ensure that the manuscript adheres to the PLOS ONE submission guidelines.
RE: Thank you for your professional advice. We have made detailed revisions according to the submission guideline. Please review our revised article.
2. References:
- Some references are missing or incorrectly formatted. Ensure that all references are complete and correctly formatted according to the PLOS ONE style guide.
RE: Thank you for your professional advice. We have checked all the references and unified all the formats into the PLOS ONE format as required. Please review our revised article.
3. Typos and Grammar:
- There are several typos and grammatical errors throughout the manuscript. A thorough proofread is recommended to improve clarity and readability.
RE: Thank you for your professional advice. We have thoroughly proofread the entire article and corrected all the grammatical errors found. Please review the revised version of our article.

Major Concerns

Study Design
- The concentration range for artesunate (30-480 μmol/L) is quite wide and requires justification based on physiologically achievable levels.
RE: Thank you for your professional advice. We have set the concentrations more meticulously and conducted CCK8 experiments on MCF7 cell lines to detect the effectiveness of concentrations. Regarding the discussion of concentrations, experimental requirements, and the review of similar experiments in the literature, we conducted a detailed investigation and reference. We also performed detailed and meticulous concentration tests over a smaller range in preliminary experiments and carried out corresponding experiments under physiological conditions.

- Control experiments using non-malignant breast cells (MCF10A) should be more extensively described.
RE: Thank you for your professional advice. For the detection of cancer cells, MCF10A, a normal epithelial cell line, is commonly used. MCF10A cells grow relatively slowly. When they reach a certain stage, their growth is contact-inhibited, forming a monolayer of cells and expressing markers of normal epithelial cells. If artesunate shows a significant inhibitory effect on MCF7 while having no obvious toxic effect on MCF10A, it indicates that artesunate has great potential for drug development. And it is explained in detail in the Results 1 section.
Technical Issues
- The Western blot methodology lacks details about loading controls and quantification methods.
RE: Thank you for your professional advice. We have already incorporated your suggestions into the evaluation of the raw data, and we have included control groups for each comparative experiment in the figure legends, which is essential. Additionally, we have provided a detailed guide on how to perform semi-quantitative analysis using ImageJ. Launch ImageJ and open your Western Blot image file via File > Open. Convert the image to an 8-bit grayscale image by selecting Image > Type > 8-bit. Use the Rectangle Tool or Freehand Selection Tool from the toolbar to select the band regions you wish to analyze. After selecting the bands, measure the grayscale values of the selected regions by choosing Analyze > Measure (or simply press Ctrl+M). The measurement results, including Area, Mean, Max, and Min, will be displayed in the Results window. Export the data from the Results window to an Excel or CSV file for further analysis using GraphPad Prism 8.
- The plasmid transfection efficiency validation data is not presented.
RE: Thank you for your professional advice. Before starting the experiment, we verified the efficiency of plasmid transfection. We found that in the MCF7 and HCC1395 cell lines, compared with the Vector group, the expression efficiency of TUG1 mRNA in the TUG1-OE group was significantly increased. Moreover, in both MCF7 and HCC1395 cells, compared with the si-NC group, the mRNA expression levels of TUG1 in the si-TUG1-1, si-TUG1-2, and si-TUG1-3 groups were significantly reduced. This indicates the effectiveness and rationality of the transfection and lays a foundation for the subsequent experiments.

Data Presentation
- Statistical analyses methods are not clearly described.
RE: Thank you for your professional advice. We have placed the statistical methods section in the figure legend. Please review it.
- Some experimental replicates numbers are not specified.
RE: Thank you for your professional advice. We have placed the number of experimental repetitions in the figure legend. Please review it.


Recommendations

1. Include dose-response curves for artesunate treatment
RE: Thank you for your professional advice. We investigated the inhibitory effects of artesunate at different concentrations on the breast cancer cell lines MCF7 and HCC1395. Moreover, we plotted the concentration-cell inhibition rate response curve, which is shown in the figure below.

2. Add detailed statistical analysis methods
RE: Thank you for your professional advice. We have added the statistical analysis method to the legend section.
3. Provide quantification for Western blot data

RE: Thank you for your professional advice. We have provided the quantitative analysis method for the same issues mentioned above. Please refer to the Raw_data file for details.
4. Validate key findings using additional breast cancer cell lines
RE: Thank you for your professional advice. We have used another breast cancer cell line, HCC1395, to conduct experiments including the CCK8 assay, EdU assay, scratch assay, Transwell invasion assay, and TUNEL assay to investigate the effects of artesunate on the proliferation, migration, invasion, and apoptosis of breast cancer cells.Please refer to Figures 1, 2, 3, and 4 for details.

5. Include transfection efficiency data

RE: Thank you for your professional advice. We have provided the results of the validation experiments for transfection efficiency. As answered in the above question.

Reviewer #2:

1. Justify dose selection based on pharmacokinetic data or previous in vivo studies to ensure translational relevance.

RE: Thank you for your professional advice. We have referred to the data in the article "Population pharmacokinetics of artesunate and dihydroartemisinin during long-term oral administration of artesunate to patients with metastatic breast cancer." This article uses a first-order absorption model. According to the full concentration-time curve data, the clearance rate of artesunate is calculated to be 35.0 L/h. We have determined that patients with metastatic breast cancer will receive oral treatment with 100 mg, 150 mg, or 200 mg of ARS once a day respectively. According to the article "Biomarker Profiling Revealed Carcinoembryonic Antigen as a Target of Artesunate in a Ductal Breast Cancer Patient", a dose of 150-300 mg for treatment can keep the patient's condition stable for about 1.5 years. According to the article "Long-term add-on therapy (compassionate use) with oral artesunate in patients with metastatic breast cancer after participating in a phase I study (ARTIC M33/2)", among 13 patients with metastatic breast cancer, long-term treatment with up to 200 mg of artemisinin (ART) orally per day (at a dose of 2.3-4.1 mg/kg body weight/day) was carried out, with a maximum cumulative treatment duration of 1115 days (37 months), and no major safety issues were triggered. Based on relevant animal experiment data, according to the article "Antitumor effects of artesunate on human breast carcinoma MCF-7 cells and IGF-IR expression in nude mice xenografts", artesunate (ART) was used at two doses: the low-dose group: 100 mg/kg/day and the high-dose group: 200 mg/kg/day. The tumor inhibition rate of the high-dose group was significantly higher than that of the low-dose group. In the nude mouse tumorigenesis experiment, the optimal effective concentration of artesunate is 200 mg/kg/day. Oral administration once a day can significantly inhibit tumor growth. According to the above concentrations, after conversion based on body surface area and drug bioavailability, the dose unit used in in vitro experiments is 421 μM. We use this concentration as a relatively high experimental concentration in in vitro experiments, which has a certain reference value, and we have also obtained data on the effects of low, medium, and high concentrations.

2. Include details on assay validation and reproducibility to strengthen methodological rigor.

RE: Thank you for your professional advice. The design of each experiment was biologically repeated three times, which makes the results more reproducible. Moreover, the original figures were rigorously used for statistical analysis, and the statistical analysis methods used are described in the figure legends. Please refer to the figure legends for details.
3. Conduct rescue experiments (e.g., over-expression or inhibition of miR-145-5p/HOXA5) to confirm the pathway’s role in artesunate’s effects.
RE: Thank you for your professional advice. Based on the original experimental design, over-expression experiments of miR-145-5p and inhibition experiments of HOXA5 have been added. First, RT-PCR experiments were conducted to verify the expression levels of three RNAs, namely TUG1, miR-145-5p, and HOXA5. Then, tests such as cell malignant behavior proliferation experiments, scratch tests, invasion experiments, and apoptosis experiments were carried out. The results of these experiments are presented as part of Result 6&7 and written in the main text. The following results are plotted as a result of performing downstream gene miR-145-5p/HOXA5 remedy.

4. Include in vivo experiments (e.g., xenograft mouse models) to validate the translational potential of artesunate.

RE: Thank you for your professional advice. Undoubtedly, conducting in vivo experiments would enhance the reliability of our research conclusions. However, considering the current limitations of laboratory resources and time, our primary focus will remain on in vitro studies in the foreseeable future. Looking ahead, we intend to carefully consider your valuable suggestion, incorporate nude mouse tumor experiments into our research plan, and establish stable cell lines to construct in vivo models, so as to further clarify the oncogenic potential of the cells. We sincerely appreciate your understanding and consistent support.
5. Add an ethics statement regarding the sourcing and use of cell lines, as per PLOS ONE guidelines.

RE: Thank you for your professional advice. We purchased commercially available cell lines. This article does not involve animal experiments or human experiments, so the explanation is relatively brief. These cells were commercial cell lines, all of which were purchased from Procell Life Science & Technology Co., Ltd. and had undergone Short Tandem Repeat (STR) identification. Below is the STR profiling report for MCF7 and MCF10A.

6. The introduction contextualizes artesunate well but could benefit from more discussion on its pharmacokinetics and potential side effects. I recommend that authors include a brief discussion on artesunate’s pharmacokinetics, toxicity, and challenges in clinical application.

RE: Thank you for your professional advice. We have searched the relevant literature on pharmacokinetics, toxicity, and clinical applications. Due to the confidentiality of the research, there are limited articles on the pharmacokinetics of artesunate in cancer treatment. One of the articles, "Population pharmacokinetics of artesunate and dihydroartemisinin during long-term oral administration of artesunate to patients with metastatic breast cancer," describes that artesunate is rapidly metabolized in vivo to dihydroartemisinin (DHA), which is its primary active metabolite. DHA is further metabolized via glucuronidation by UDP-glucuronosyltransferases, forming α-DHA-β-glucuronide, the major metabolite of DHA. The elimination half-life of DHA is relatively short, ranging from 0.5 to 1.5 hours. In patients undergoing long-term (over 3 weeks) oral artesunate therapy, a 24.9% increase in the apparent oral clearance of DHA has been observed. Artesunate, administered orally, intravenously, or intramuscularly, is rapidly distributed throughout the body, with particularly high concentrations in the liver, spleen, and tumor tissues.

Regarding the hepatotoxicity of artesunate during cancer treatment, based on reports from "Hepatotoxicity by combination treatment of temozolomide, artesunate and Chinese herbs in a glioblastoma multiforme patient: case report review of the literature" and "Fatal Liver and Bone Marrow Toxicity by Combination Treatment of Dichloroacetate and Artesunate in a Glioblastoma Multiforme Patient: Case Report and Review of the Literature," artesunate may cause severe liver damage and bone marrow suppression when used in combination with dichloroacetate (DCA) or traditional Chinese herbs.

Clinically, artesunate may also exhibit certain side effects. At low doses, it may demonstrate anti-angiogenic effects, while at higher doses, it may directly kill tumor cells. However, precise dose control is essential to avoid toxic side effects. When combined with other anticancer drugs (such as temozolomide or doxorubicin) or traditional Chinese herbs, artesunate may induce unpredictable toxic reactions. Although artesunate has demonstrated significant anticancer activity in vitro and in animal models, tumor cells may develop resistance through various mechanisms, such as alterations in DNA repair pathways, in clinical applications. Currently, the use of artesunate in cancer therapy is primarily based on small-scale clinical trials and case reports, and there is a lack of large-scale, multicenter randomized controlled trial data to validate its safety and efficacy.

We have incorporated the above content into the discussion section.
7. The figures could be clearer, kindly provide clearer versions of them.
RE: Thank you for your professional advice. We have provided a clearer version. We’ve made detailed modifications and color adjustments to each picture, and adjusted the image from 300 DPI to 600 DPI.

8. Elaborate on limitations such as the in vitro nature of the study, lack of long-term toxicity data, and absence of patient-derived models in the limitations section.

RE: Thank you for your professional advice. The limitations of this study indeed lie in its focus on the role of Artesunate in regulating the malignant phenotypes of breast cancer cell lines through the LncRNA TUG1 target. Further exploration and research are needed to understand how Artesunate functions in vivo and how it can be applied in clinical settings. The study employed various concentrations of Artesunate in experiments, but it did not clearly specify whether these doses are clinically feasible in vivo. This indicates that the research was primarily conducted in vitro, lacking support from in vivo experimental data. While in vitro experiments are useful for preliminary exploration of drug mechanisms, their results may not fully reflect the true effects of the drug in the complex biological environment of the body, which involves various physiological and metabolic factors. High doses of Artesunate may induce toxicity in normal cells, potentially limiting its clinical application. However, the study did not provide data on the long-term toxicity of Artesunate. Long-term toxicity data are crucial for assessing the safety and potential side effects of a drug, especially in cancer treatment where patients may require prolonged medication. The study also did not explicitly mention the use of patient-derived tumor models (such as patient-derived xenograft models, PDX models). These models are better able to simulate the heterogeneity and complexity of human tumors and are important tools for evaluating drug efficacy and toxicity. If the study relied solely on standardized cell lines, its results may not adequately reflect the drug's effects in real patients. Therefore, the absence of patient-derived models could be a significant limitation of this study.
9. Reframe the conclusion to emphasize the need for further in vivo and clinical studies.

RE: Thank you for your professional advice. We have added content about the necessity of conducting in vivo and clinical studies in the discussion section.
In conclusion, this study demonstrates that artesunate has the potential to modulate the malignant progression of breast cancer cells through the lncRNA TUG1/miR-145-5p/HOXA5 axis, offering promising evidence for its anti-cancer effects. However, the findings are primarily based on in vitro experiments, which may not fully capture the complexity of biological systems in vivo. To advance this research toward clinical application, further studies are imperative. These should include in vivo experiments to validate the efficacy and safety of artesunate in animal models, as well as clinical trials to assess its feasibility, optimal dosing, and long-term toxicity in human patients. Additionally, the use of patient-derived models, such as patient-derived xenografts (PDX), could provide more accurate insights into the drug’s therapeutic potential in real-world scenarios. Addressing these gaps will be critical to translating these promising findings into effective and safe breast cancer therapies.
10. Including computational modelling (e.g., molecular docking) to explore artesunate’s interaction with lncRNA TUG1 and related targets will be advantageous.

RE: Thank you for your professional advice. After querying the databases and relevant knowledge about molecular docking experiments, We’ve learned that molecular docking refers to the formation of a three-dimensional imaging model between a drug monomer and a protein molecule. The main topic of our article is about how the extracellular drug artesunate regulates the intracellular long non-coding RNA (LncRNA), which does not meet the requirements of the database for drug-protein interactions. Moreover, most of the existing molecular docking software is developed for protein-ligand interactions, and its applicability to drug-lncRNA interactions is limited. It’s possible that the relevant databases are still under development. We will continue to follow the content in this research field and gradually improve the operation techniques of such software.

6. PLOS authors have the option to publish the peer review history of their article ([what does this mean?](https://track.editorialmanager.com/CL0/https://journals.plos.org/plosone/s/editorial-and-peer-review-process%23loc-peer-review-history/1/010f019536613acf-1663e915-de07-4821-a194-1d2656418e62-000000/6Z98Rb3atkRLmUEdb6ISstUSqP5EjL5dlvY-RZIU9UY=199)). If published, this will include your full peer review and any attached files.
If you choose “no”, your identity will remain anonymous but your review may still be made public.
**Do you want your identity to be public for this peer review?** For information about this choice, including consent withdrawal, please see our [Privacy Policy](https://track.editorialmanager.com/CL0/https://www.plos.org/privacy-policy/1/010f019536613acf-1663e915-de07-4821-a194-1d2656418e62-000000/Roa27MLL3cN2gcDK70LEF7BKxVYiYSokDo6hgCnFtRo=199).

Reviewer #1: No

Reviewer #2: No

[NOTE: If reviewer comments were submitted as an attachment file, they will be attached to this email and accessible via the submission site. Please log into your account, locate the manuscript record, and check for the action link "View Attachments". If this link does not appear, there are no attachment files.]

While revising your submission, please upload your figure files to the Preflight Analysis and Conversion Engine (PACE) digital diagnostic tool, [https://pacev2.apexcovantage.com/](https://track.editorialmanager.com/CL0/https://pacev2.apexcovantage.com//1/010f019536613acf-1663e915-de07-4821-a194-1d2656418e62-000000/ypmgaUGPdPSvMEjpw228jHjKvsWp9Rvi7gdyqbIPeG0=199). PACE helps ensure that figures meet PLOS requirements. To use PACE, you must first register as a user. Registration is free. Then, login and navigate to the UPLOAD tab, where you will find detailed instructions on how to use the tool. If you encounter any issues or have any questions when using PACE, please email PLOS at [figures@plos.org](mailto:figures@plos.org). Please note that Supporting Information files do not need this step.
